# Impact of Acute Surgical Assessment Unit Dedicated Ultrasound Access at a Tertiary Care Hospital

**DOI:** 10.7759/cureus.17294

**Published:** 2021-08-18

**Authors:** Muhammad Fahad Ullah, Rishabh Sehgal, Christina Fleming, Shiori Kimura, Laura Rouche, Shona Tormey

**Affiliations:** 1 General Surgery, University Hospital Limerick, Limerick, IRL

**Keywords:** surgical assessment unit, ultrasound, quality improvement

## Abstract

Background

Delayed diagnosis, unnecessary hospital admissions and extended length of stay are the problems associated with inappropriate access to radiological investigations. The acute surgical assessment unit (ASAU) in Limerick has two dedicated ultrasound scan (USS) slots daily to overcome this problem. The aim of the current study was to investigate the clinical impact on patient care and the cost-effectiveness of such an ASAU USS access.

Methods

A retrospective review of all patients who underwent USS investigation in the ASAU between May and September 2017 was conducted. Demographic, referral source, presenting complaint, and clinical outcome data were obtained from the ASAU Log. USS data was obtained from the National Integrated Medical System (NIMIS). The Integrated Patient Management System (IPMS) and Therefore Case Manager, Therefore 2014(12.0.2) was utilized to check for any discharged ASAU patient re-presenting to the emergency department (ED) within 30 days.

Results

A total of 102 patients underwent USS investigation during the study period. The most common presenting complaint was epigastric or right upper quadrant pain (55.8%). Eighty-six patients underwent USS on the same day and the majority (51%) were discharged home with appropriate outpatient follow-up. Approximately 26,000 Euros were saved over four months. Post-discharge ED visits in the ASAU discharged group was zero in the 30 days.

Conclusion

The ASAU USS dedicated slots in University Hospital Limerick has had a significant positive impact on patient diagnostics, surgical admissions rates and streamlining resource allocation. Having dedicated slots for radiological investigations in the ASAU should become standard of care across all healthcare jurisdictions.

## Introduction

In 2009 Mid-Western Regional Hospitals Group underwent reconfiguration to improve patient safety and streamline provision and costs. The centralisation of all emergency surgery to University Hospital Limerick (UHL) resulted in long waiting times in the emergency department (ED) and increased hospital admission rates. One solution to this was the establishment of an acute surgical assessment unit (ASAU), a dedicated area where acutely ill surgical patients can be assessed and monitored prior to being admitted to the hospital or being treated and discharged. ASAU forms an integral part of the National Clinical Programme (NCPS) 2013 ‘Model of Care for Acute Surgery’ and aims to streamline category 3 and 4 patients (Manchester Triage System, 2014) flow and optimizes resource allocation in order to provide timely access to assessment, investigation and senior decision making.

Ultrasound scan (USS) is a common, non-invasive and relatively cheap investigation for emergency surgical referrals. Difficulty in obtaining timely access to radiological investigations can lead to delay in diagnosis and focused management plans. Prior to 2014, a large proportion of surgical referrals were admitted to UHL in order to undergo USS the next day. Such delays in diagnostics led to unnecessary hospital admissions and extended length of stay. This had a significant knock on implications on clinical, cost and service provision.

The ASAU in UHL has two dedicated slots for ultrasound daily. The aim of the current study was to study the clinical impact of ASAU ultrasound dedicated slots on patient care and cost-effectiveness. 

This article was published as an abstract/poster in https://map.amegroups.com/article/view/4785/html.

## Materials and methods

A retrospective review of all patients who underwent USS from the ASAU in UHL was performed between May and September 2017. Patients were identified from the ASAU log that is prospectively maintained by the Clinical Nurse Manager (CNM). This log contains salient data pertaining to the referral source, demographics, presenting complaint, investigations, management plan and outcome(whether the patient was discharged home or admitted) for all patients attending the ASAU. USS data was obtained from the hospital National Integrated Medical System (NIMIS). The indications were recorded from the ASAU log and requests were checked on NIMIS for each patient. Final reports by radiologists were noted from NIMIS and the decision to admit or discharge with a follow-up plan was noted from the ASAU log. Only those ultrasounds that were booked by ASAU registrars were taken into account. The Integrated Patient Management System (IPMS) and Therefore case manager (2014 version 12.0.2) was utilised to check for same-day discharged ASAU patient representing to the ED within 30 days. Hospital day admission charges and ultrasound abdomen charges were obtained from Health Service Executive website. Data were collected and analysed on Microsoft Office Excel spreadsheet (2007). A cost-effectiveness analysis was also carried out to look at the sustainability and benefits of the service. The project was approved by institutional audit committee.

## Results

A total of 102 patients that attended the ASAU underwent USS during the study period. Demographic data are summarized in Table 1. The majority of patients were female (82%) with a mean age of 43±18.7 years. USS was performed on the same day in 84.3% of cases (N = 86). Out of these, 51.1% were discharged home with appropriate outpatient follow-up organized. The remainder were admitted to the hospital. Sixteen patients underwent USS on a later date out of which only one patient (6.25%) was subsequently admitted.

**Table 1:** Demographics. N = number. *Where applicable.

	%*	N*
Mean age ± SD (years)	43 ±18.7	
M/F	21.56/78.4	22/80
Distribution as per presenting complaints		
Biliary system-related complaints	55.8	48
Right iliac fossa pain	25.6	22
Lower abdomen/left iliac fossa/flank pain	9.3	8
Mass/swelling	9.3	8
Hospital admission and discharge post ultrasound		
				% of patients	N*
Ultrasound on same day	84.3	86
Discharged on same day	51.1	44
Ultrasound on a later date	15.6	16
Admitted to hospital (out of 16)	6.25	1

**Figure 1:**
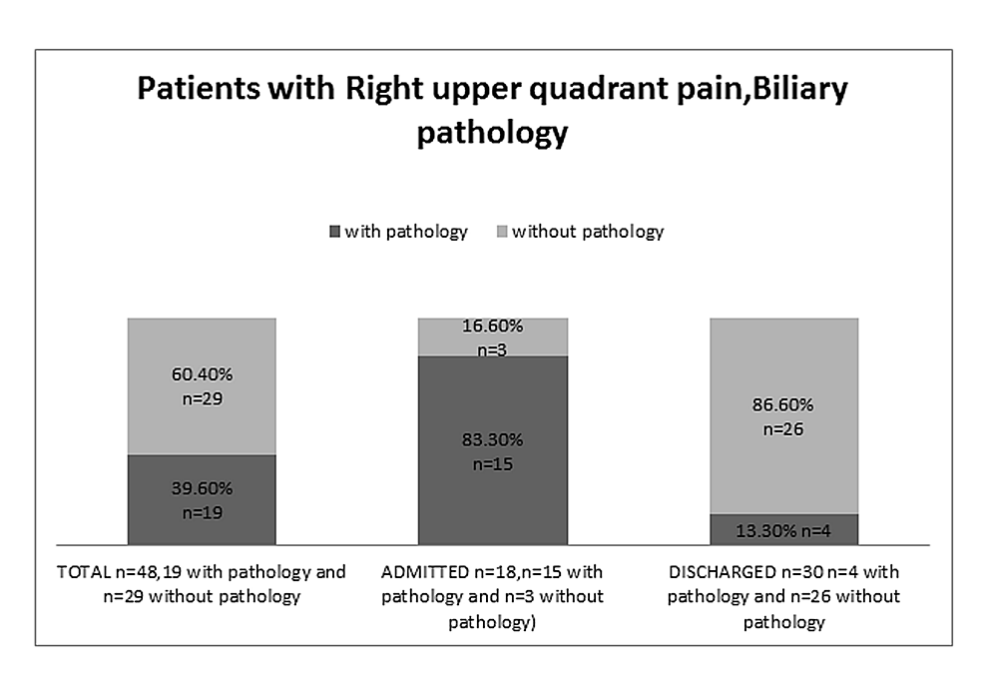
Ultrasound results and outcomes of patients with biliary-related complaints.

Out of the 86 patients who received ultrasound on the same day, 48 (55.8%) had biliary-related symptomatology and 22 (25.6%) presented with right iliac fossa (RIF) pain. The remainder had non-specific generalized abdominal pain or presented with abdominal masses or swellings (Table 1). Out of the 48 patients with biliary type symptoms, USS led to 18 (37.5) acute surgical admissions while 62.5% (n = 30) were discharged home. USS failed to demonstrate any pathology in 60.4% of these 48 patients and diagnosed benign pathology in 39.6% (Figure 1).

**Figure 2:**
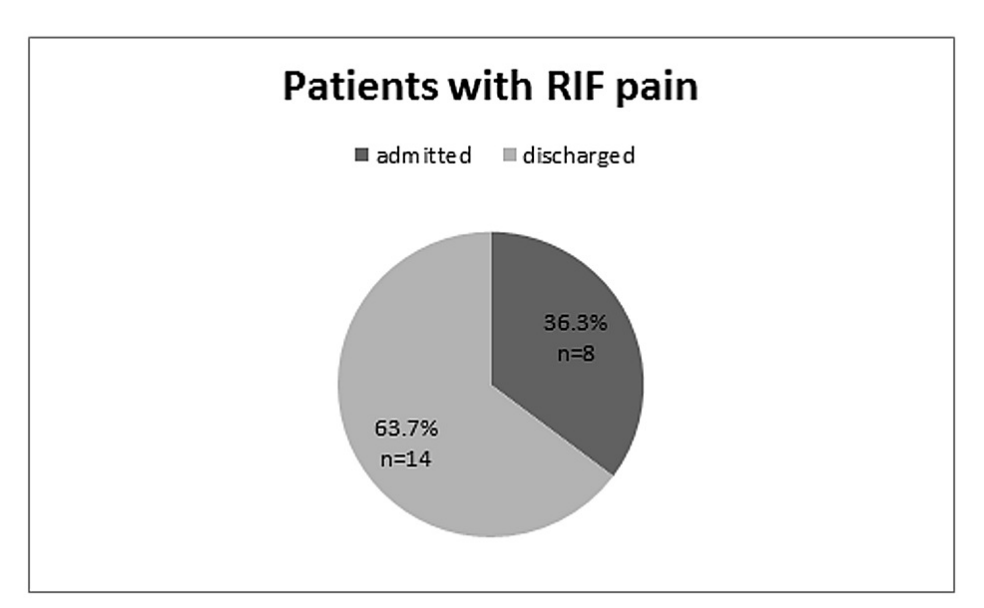
Proportion of patients with RIF pain admitted and discharge. RIF: right iliac fossa.

The overall USS findings for the study cohort is summarized in Table 2. Twenty-two patients (25.6%) who underwent USS on the same day were referred with right iliac fossa pain. Out of this group, 36.3% (N = 8) were admitted to the hospital and 14 (63.7%) were discharged home (Figure 2). A minority of patients (9.3%, n = 8) who had USS on the same day, had symptoms of lower abdominal or left iliac fossa pain. Another group was patients referred with an abdominal mass or swelling (9.3% of the total cohort, N = 8). The majority of the scans among these were normal (62.5%, N = 5/8).

**Table 2:** Ultrasound findings in different request groups. N = number. LIF: left iliac fossa; RIF: right iliac fossa.

Ultrasound findings for patients with RIF pain, N = 22		
Findings	N = 22	%
		(out of N = 22)
Normal	12	54.5
Biliary system involvement	6	27.3
Ovarian pathology	2	9
Pancreatitis	1	4.5
Appendicitis	1	4.5
Ultrasound findings in patients with lower abd/LIF/flank pain		
Ultrasound findings	N=8	% (out of 86)
Ovarian mass	2	2.3
Dilated cbd/Choledocholithiasis	2	2.3
Appendicitis	1	1.1
Acute cholecystitis	1	1.1
Normal	1	1.1
Gall bladder polyp	1	1.1
Ultrasound findings in patients with masses or swelling, as request indication		
Ultrasound findings	N = 8	%
		(out of 86)
Normal	5	5.8
Free fluid	1	1.1
Biliary system-related findings	2	2.3

Six patients (14.3%) who were admitted from the ASAU represented to ED within 30 days from the date of discharge. No patients discharged directly from the ASAU represented to the ED within 30 days. Three patients who were discharged from the ASAU represented to the ED at three months, one patient was admitted and two were discharged. One patient represented to the ED at six months and was discharged home.

## Discussion

University Hospital Limerick is a Model 4 hospital in the Mid-Western area of Ireland. It is part of the Mid-Western Regional Hospitals Group incorporating Limerick (general and maternity), Ennis, Nenagh, St. Johns and Croom hospital. Before 2009, each of these hospitals offered ED and intensive care services with medical and surgical teams on site. To improve patient safety and streamline health provision and costs, the surgical services in the Mid-West Area underwent reconfiguration in 2009 following the recommendations outlined by the Health Service Executive (HSE) Teamwork-Howath Report on acute services in the Mid-West Area [1]. All surgical referrals from the three peripheral hospitals are redirected to UHL for assessment. This has resulted in an overwhelmingly high volume of surgical referrals to an already overburdened ED. Potential solutions to offsetting this real problem included the establishment of a dedicated emergency theatre, the ‘surgeon of the week’ model and the ASAU.

The concept of an ASAU has been advocated by many healthcare systems since the mid-1990s. This was based on the increasing numbers of emergency surgical patients not meeting prescribed Patient Experience Times within the ED. Although Category 1 and 2 patients (Manchester Triage System) continue to be assessed through the ED, the ASAU provides an ideal environment for the efficient assessment of Category 3 and 4 patients. The ASAU in UHL provides a facility that allows the general practitioners (GPs) a direct access to emergency surgical services. Johnstone et al. recently demonstrated the safety and effectiveness of primary care referrals to a surgical clinical decision unit [2].

As per the National Clinical Programme (NCPS) 2013 ‘Model of Care for Acute Surgery’, ASAU forms an integral part of acute surgical services and aims to streamline category 3 and 4 patients (Manchester Triage System, 2014) flow. Acute SAU also optimises resource allocation in order to provide timely access to assessment, investigation and senior decision making [3]. This project was done in 2018 and presented as oral presentation at Sylvester O'Halloran surgical conference 2019 and the abstract was published in Mesentery peritoneum 2019 [4].

The ASAU in UHL has access to two dedicated USS slots per day. USS is a common, cheap and non-invasive radiologic modality utilized to investigate surgical referrals. It has been demonstrated that USS is valuable in making the correct diagnosis during the initial evaluation of patients presenting with acute abdominal pain. Moreover, the concordance of USS findings with the discharge diagnosis is high [5]. Dhillon et al. reported on the therapeutic impact of abdominal USS in patients with acute abdominal symptoms and showed that the diagnostic confidence significantly increased following USS. USS was valuable in tailoring management as the intended management plan changed in 22 patients, fifteen intended laparotomies were halted and a further seven patients underwent surgery where this was not originally intended. In this study USS was rated either 'very' or 'moderately' helpful in 87% of patients, with 99% of clinicians finding it either 'very' or 'moderately' helpful generally [6].

A sizeable proportion of surgical referrals are admitted due to limited access to diagnostics. This can lead to delayed diagnosis, patient management, and increased length of stay (LOS). Lai et al. recently reported on the value of their surgical assessment unit ultrasound facility in 100 patients. In this retrospective study, ASAU USS resulted in a reduced LOS by 1.44 days and led to more same-day discharges thus avoiding unnecessary hospital admissions. Furthermore, the study demonstrated a significant reduction in mean waiting times from admission to investigation by 5.21 hours, which translated into improved patient and staff satisfaction [7]. Similarly, Tierney et al. recently reported on their experience in the management of surgical emergencies that underwent an emergency surgery ambulatory care pathway. Abdominal and pelvic USS was available in the SAU in dedicated slots each morning. In this study, 69% patients suitable for the emergency ambulatory care pathway avoided admission. They reported a high level of satisfaction by both patients and GPs. This four-month pilot study translated to an annual savings of £1.34 million to the healthcare system [8]. These results are in line with our study whereby same-day USS resulted in the majority of ASAU patients (51%) to be discharged home with appropriate follow-up. Furthermore, our study did not demonstrate any readmissions within 30 days in USS patients who were discharged directly from the ASAU.

There is a high prevalence of patients consulting GPs for abdominal pain. A recent systematic review of symptom-evaluating studies on prevalence, aetiology or prognosis of abdominal pain in the primary care setting demonstrated that in approximately one-third of patients the underlying cause of abdominal pain cannot be specified. The most common aetiologies for abdominal pain in this robust systematic review included gastroenteritis (7.2%-18.7%), irritable bowel disease (2.6-13.2%), urological cause (5.3%) and gastritis (5.2%). Crucially, one in 10 abdominal pain patients suffers from an acute disease such as appendicitis (1.9%), diverticulitis (3.0%), biliary/pancreatic (4.0%) or neoplastic (1.0%) diseases necessitating immediate medical attention [9]. This high rate of acute underlying diseases warrants further investigation or therapy and as such the ASAU provides GPs and patients direct access to prompt surgical evaluation.

In our study, the most common presenting complaint was right upper quadrant abdominal pain which was most likely biliary in origin. Acute cholecystitis can be life-threatening therefore accurate and early diagnosis is important to initiate appropriate and timely management. Trowbridge et al. performed an extensive systematic review to investigate whether aspects of the history and physical examination or basic laboratory testing clearly identified patients who required diagnostic imaging investigations to establish a reliable diagnosis of acute cholecystitis. After analysing 17 studies, no single clinical finding or laboratory test carried sufficient weight to establish or exclude cholecystitis without further testing (e.g., right upper quadrant USS) [10]. Furthermore, Allemann et al. prospectively evaluated the routine use of abdominal USS in patients presenting to the surgical emergency unit with acute abdominal pain over a 12-month period. USS improved the correct diagnostic rate from 70% to 83%. The diagnostic accuracy for acute appendicitis and biliary tract disease improved after USS from 92% to 98% and from 93% to 99% respectively [11]. In our study cohort, the ASAU USS facility led to 37.5% surgical admissions in patients presenting with RUQ pain with the remainder being discharged home the same day. Moreover, USS failed to show any pathology in 60.4% of cases with RUQ pain and 54.5% in RIF pain patients. This is in agreement with the study by Grubel who evaluate the utility of gastroenterologist operated abdominal USS in a community practice. In this study, USS did not detect any pathologies in almost half of the cases [12]. Walsh et al. showed in a prospective randomized controlled trial that selective ultrasound improved diagnostic yield compared to immediate ultrasound and is believed to be the better option. It implies that a prompt but selective ultrasound service should be available for in-patients in the acute specialties. This explains our two dedicated slots availability that makes practitioners choose the appropriate patient for undergoing USS. Efforts are made to use ultrasound slots wisely for those patients in whom a decision is strengthen by USS [13].

The current study is limited by the fact that it contains a small study cohort performed over a four-month period only. Moreover, the data is largely descriptive in nature. A single abdominal USS abdomen costs approximately 100 euros, while hospital admission costs approximately 800 Euro/day. This translates to an estimated saving of approximately 700 Euro per patient, and 26,000 Euro over the four-month study period. This can be extended to an annual cost savings of approximately 75,000 Euros to the Irish health service.

A potential future development to the ASAU USS facility would be to extend the current successful weekday service to weekends to achieve the benefits of same-day discharges and short length of stay. This would resolve the ‘bed-blocking’ issue that occurs during the weekends as patients are often waiting for semi-urgent radiological investigations that take place the following week, this subsequently could also free up beds for elective patients to come in thus avoiding potential postponement of surgeries. Appropriate resource and staff allocation would be warranted in order for any further development to this service.

## Conclusions

The ASAU USS access in UHL has successfully been implemented since the reconfiguration of services in the Midwestern region of Ireland. The service has proven effective in patient diagnosis and management. It avoids unnecessary admissions. It helps in improving patient flow providing financial benefits thus we advocate flexible USS slots to be part of every modern ASAU.
